# Cosmosiin Increases ADAM10 Expression via Mechanisms Involving 5’UTR and PI3K Signaling

**DOI:** 10.3389/fnmol.2018.00198

**Published:** 2018-06-11

**Authors:** Zhuo Min, Ying Tang, Xiao-Tong Hu, Bing-Lin Zhu, Yuan-Lin Ma, Jing-Si Zha, Xiao-Juan Deng, Zhen Yan, Guo-Jun Chen

**Affiliations:** ^1^Department of Neurology, The First Affiliated Hospital of Chongqing Medical University, Chongqing Key Laboratory of Neurology, Chongqing, China; ^2^Department of Neurology, The Ninth People’s Hospital of Chongqing, Chongqing, China; ^3^Department of Physiology and Biophysics, State University of New York at Buffalo, Buffalo, NY, United States

**Keywords:** cosmosiin, ADAM10, translation, 5’UTR, PI3K

## Abstract

The α-secretase “a disintegrin and metalloproteinase domain-containing protein” (ADAM10) is involved in the processing of amyloid precursor protein (APP). Upregulation of ADAM10 precludes the generation of neurotoxic β-amyloid protein (Aβ) and represents a plausible therapeutic strategy for Alzheimer’s disease (AD). In this study, we explored compounds that can potentially promote the expression of ADAM10. Therefore, we performed high-throughput small-molecule screening in SH-SY5Y (human neuroblastoma) cells that stably express a luciferase reporter gene driven by the ADAM10 promoter, including a portion of its 5’-untranslated region (5’UTR). This has led to the discovery of cosmosiin (apigenin 7-O-β-glucoside). Here, we report that in human cell lines (SH-SY5Y and HEK293), cosmosiin proportionally increased the levels of the immature and mature forms of the ADAM10 protein without altering its mRNA level. This effect was attenuated by translation inhibitors or by deleting the 5’UTR of ADAM10, suggesting that a translational mechanism was responsible for the increased levels of ADAM10. Luciferase deletion assays revealed that the first 144 nucleotides of the 5’UTR were necessary for mediating the cosmosiin-induced enhancement of ADAM10 expression in SH-SY5Y cells. Cosmosiin failed to increase the levels of the ADAM10 protein in murine cells, which lack native expression of the ADAM10 transcript containing the identified 5’UTR element. The potential signaling pathway may involve phosphatidylinositide 3-kinase (PI3K) because pharmacological inhibition of PI3K attenuated the effect of cosmosiin on the expression of the ADAM10 protein. Finally, cosmosiin attenuated Aβ generation because the levels of Aβ40/42 in HEK-APP cells were significantly reduced after cosmosiin treatment. Collectively, we found that the first 144 nucleotides of the ADAM10 5’UTR, and PI3K signaling, are involved in cosmosiin-induced enhancement of the expression of ADAM10 protein. These results suggest that cosmosiin may be a potential therapeutic agent in the treatment of AD.

## Introduction

ADAM10 belongs to the type I transmembrane protease family that plays an important role in various cellular functions including neurogenesis, angiogenesis, heart development and fertilization (Moss et al., 2007; Edwards et al., 2008; Pruessmeyer and Ludwig, 2009; Jouannet et al., 2016). In the brain, the amyloid precursor protein (APP) is one of the major targets of ADAM10 (Kuhn et al., 2010). Sequential proteolytic processing of APP by β-secretase 1 (BACE1) and γ-secretase generates the pathogenic β-amyloid protein (Aβ), which plays causative role in Alzheimer’s disease (AD; De Strooper et al., 2010). Conversely, the α-secretase ADAM10 cleaves APP to form the soluble APPα (sAPPα) and α-COOH-terminal fragment (α-CTF), precluding the generation of Aβ (Postina, 2012). ADAM10 can decrease the Aβ load in mouse models of AD (Postina et al., 2004; Schroeder et al., 2009). Impaired ADAM10 trafficking generates a model of sporadic AD (Epis et al., 2010). Similarly, studies conducted with human AD patients show deficits in the expression of ADAM10 (Marcinkiewicz and Seidah, 2000). The levels of sAPPα are lower in the platelets and cerebrospinal fluid of patients with AD (Sennvik et al., 2000; Colciaghi et al., 2002; Fellgiebel et al., 2009). Upregulation of ADAM10 represents a promising therapeutic strategy for AD (Endres and Fahrenholz, 2010; Lichtenthaler, 2011).

The expression of ADAM10 can be regulated at transcriptional and translational levels (Vincent, 2016). Transcription factor PPARα (proliferator-activated receptor-α) and the vitamin A analog acitretin promote the transcription of ADAM10 via retinoic acid responsive element (Tippmann et al., 2009; Corbett et al., 2015). The 5’ untranslated region (5’UTR) of ADAM10 represses the rate of ADAM10 translation (Lammich et al., 2010, 2011). The DNA sequences responsible for the ADAM10 promoter and 5’UTR partially overlap. In the ADAM10 promoter, nucleotides −508 to −300 upstream of initiation site represent a critical region in which several transcription biding sites are located (Prinzen et al., 2005). Conversely, the 5’UTR of ADAM10 spans the first 444 nucleotides (from −444 to −1) upstream of the ADAM10 initiation codon (Lammich et al., 2010). The activity of 5’UTR and translation of ADAM10 can be regulated by derivatives of 1-methylquinolinium (Dai et al., 2015). Thus, the first 444 nucleotides may be involved in the function of either the ADAM10 promoter or the 5’UTR.

We have performed high-throughput small-molecule screening in SH-SY5Y cells, which stably express the ADAM10 promoter sequence (−508 to −30) fused with a luciferase reporter gene. Among the positive hits, the histone deacetylase inhibitor apicidin can promote the transcription of ADAM10 and reduce the generation of Aβ (Hu X. T. et al., 2017). Cosmosiin, a flavonoid found in herbal medicines including *Scutellaria baicalensis* Georgi and *Matricaria*, also enhanced luciferase activity. In this study, we found that cosmosiin increased the levels of the ADAM10 protein but not the level of ADAM10 mRNA. We further observed that the 5’UTR of ADAM10, and phosphatidylinositide 3-kinase (PI3K) signaling, were involved in cosmosiin-induced enhancement of the levels of ADAM10 protein. Cosmosiin may function in APP processing, because cosmosiin treatment increased the levels of sAPPα, and decreased those of Aβ40/42, in HEK-APP cells.

## Materials and Methods

All experimental protocols, including those associated with standard biosecurity and safety, were approved by the Chongqing Medical University.

### Chemicals and Antibodies

Cosmosiin (apigenin-7-O-glucoside) was purchased from BiobioPha (Kunming, Yunnan, China); actinomycin D (ActD), cycloheximide (CHX), chloroquine (CQ), MG132, U0126 and LY294002 were purchased from Sigma-Aldrich (St. Louis, MO, USA); 3-methyladenine (3-MA), wortmannin and 4EGI1 were purchased from Selleck (Houston, TX, USA); rapamycin was purchased from Merck (Whitehouse Station, NJ, USA); DMSO was purchased from Dinguo (Beijing, China). Cosmosiin, ActD, CHX, MG132, 4EGI1, LY294002, wortmannin and rapamycin were dissolved in DMSO to generate respective stock solutions; CQ and 3-MA were dissolved in sterile water. Subsequent dilutions were performed using culture medium.

The antibodies against ADAM10 (ab1997; 1:1000), β-amyloid converting enzyme 1 (BACE1; ab2077; 1:1000), phosphor-ERK1 (p-ERK1, T202/Y204)-phosphor-ERK2 (p-ERK2, T185/Y187; ab76299; 1:5000), and ERK1/ERK2 (ab184699; 1:5000) were purchased from Abcam (Shanghai, China). Anti-APP and COOH-terminal fragment (A8717; 1:1000) were purchased from Sigma-Aldrich (St. Louis, MO, USA). Anti-sAPPα (6E10; 1:1000) and sAPPβ (1:500) were purchased from Covance (Princeton, NJ, USA). Anti-Akt (9272S; 1:1000) and p-Akt (S473; 9271L; 1:1000) were purchased from Cell Signaling (Danvers, MA, USA). Anti-6 × His (66005–1-lg; 1:1000), anti-GAPDH (60003-2-Ig; 1:4000), and horseradish peroxidase-conjugated anti-rabbit or anti-mouse secondary antibodies (1:5000) were purchased from Proteintech (Wuhan, Hubei, China). Fluorescence-labeled secondary goat anti-rabbit antibody (Alexa Fluor 488; 1:200) was purchased from Beyotime (Shanghai, China).

### Cell Culture

Human neuroblastoma SH-SY5Y and mouse microglia N9 cells were cultured in DMEM/F12 (Thermo Fisher Scientific, Waltham, MA, USA) supplemented with 10% FBS (Thermo Fisher Scientific), 100 mg/ml streptomycin and 100 U/ml penicillin G. Human embryonic kidney HEK293 and mouse hippocampal HT22 cells were cultured in DMEM supplemented with 10% FBS, 100 mg/ml streptomycin and 100 U/ml penicillin G. HEK293-APP (HEK293 cells stably expressing full-length human APP) cells were cultured in DMEM supplemented with 10% FBS, 100 mg/ml streptomycin, 100 U/ml penicillin G and 0.2 mg/ml G418.

Primary hippocampal neurons were prepared from a neonatal mouse (C57 black/6J) at day 0; hippocampus tissues were dissected, treated with 0.25% trypsin-EDTA for 15 min at 37°C, and then mechanically dissociated. Neurons were resuspended in DMEM with 20% FBS, plated on poly-L-lysine-coated dishes and incubated for 6 h. Subsequently, the culture medium was replaced with fresh Neurobasal medium (Invitrogen) supplemented with 2% B27 (Invitrogen), 100 U/ml penicillin, 100 μg/ml streptomycin and 0.5 mM glutamine. Every 2–3 days, half of the medium was removed, replenished with fresh Neurobasal medium and incubated at 37°C under 5% CO_2_. All procedures were conducted in accordance with the Chongqing Medical University Guidelines for the Care and Use of Laboratory Animals. The protocol was approved by the Animal Research Committee of Chongqing Medical University.

### Western Blotting

Cell lysates were treated using radio immunoprecipitation Assay (RIPA) buffer (1% Triton X-100, 0.5% sodium deoxycholate, 0.1% SDS, 150 mM NaCL, 1 mM EDTA, and 50 mM Tris) supplemented with phosphatase inhibitors (Boster, Wuhan, China) and protease inhibitors (Roche, Indianapolis, IN, USA). Protein concentrations were measured using a BCA Protein Assay Kit (Dingguo, Beijing, China). Samples were separated on 10 or 15% SDS-PAGE gel and transferred to a PVDF membrane (Millipore, Billerica, MA, USA). Membranes were blocked using 5% nonfat dry milk in TBST (25 mM Tris at pH 7.4, 1.5 M NaCl, and 0.05% Tween-20) for 1 h at 24°C and probed with individual antibodies overnight at 4°C. Blots were washed and incubated for 1 h with horseradish peroxidase-conjugated anti-rabbit or anti-mouse secondary antibodies. The bands were visualized using an ECL reagent (Thermo, Marina, CA, USA) and Fusion FX5 image analysis system (Vilber Lourmat, Marne-la-Vallee, France). Relative protein expression levels were calculated using Quantity One software (Bio-Rad, Hercules, CA, USA) with normalization to the signal of GAPDH.

### Immunofluorescence

Cells incubated on coverslips were washed with ice-cold PBS and fixed with 4% paraformaldehyde for 30 min at 37°C. Cells were then permeabilized in 0.3% Triton X-100 for 20 min at 24°C and incubated with the primary antibody against ADAM10 (1: 200) in 4% BSA for 3 h. Coverslips were washed with PBS three times and incubated with fluorescence-labeled secondary goat anti-rabbit antibody Alexa Fluor 488 (1:200) for 1.5 h at 24°C. After washing, the coverslips were mounted with DAPI Fluoromount-G (Southern Biotech, Birmingham, Alabama, USA). Images were acquired using a laser scanning confocal microscope (Leica TCS SP8 X, Germany). Quantification of immunofluorescence intensity was performed using Image-Pro Plus 6.0 software (Media Cybernetics, Silver Springs, MD, USA).

### Plasmid Constructs and Luciferase Assay

Human ADAM10 constructs with full-length 5’UTR and without the 5’UTR were kindly provided by Dr. Sven Lammich at Ludwig-Maximilians-University. The 5’UTR construct deleting the first 144 nucleotides was subcloned into pcDNA6/V5-His A (Invitrogen, Carlsbad, CA, USA) as described previously (Lammich et al., 2010). Human genomic DNA was extracted from cultured cells with Mammalian Genomic DNA Miniprep kit (Dinguo, Beijing, China) and was used as a template for amplification of nucleotides −467 to −30 (pGL4.17-ADAM10-D), −444 to −30 (pGL4.17-ADAM10-C), −508 to −300 (pGL4.17-ADAM10-E), −467 to −300 (pGL4.17-ADAM10-E1), −444 to −300 (pGL4.17-ADAM10-E2), and −300 to −30 (pGL4.17-ADAM10-F). PCR-amplified fragments were then cloned into the firefly luciferase reporter vector pGL4.17 (Promega, Madison, WI, USA) using primer sequences (NM_001110) listed in Table 1. Cells were transfected using Lipofectamine 3000 (Invitrogen). Luciferase activity was measured after 36 h with a GloMax 96 microplate luminometer (Promega).

**Table 1:** Primer sequences for ADAM10 constructs.

Gene	Primer sequence, 5’-3’	Restriction site	Size (bp)
*ADAM10*-C	CGGGGTACCGCGGCGGCAGGCCTAGC	*Kpn*I	415
	CCGCTCGAGTCCTCACGGGTTAACAGCAGCACAT	*Xho*I	
*ADAM10*-D	CGGGGTACCAGCTCTCCGCCGGCGGAC	*Kpn*I	438
	CCGCTCGAGTCCTCACGGGTTAACAGCAGCACAT	*Xho*I	
*ADAM10*-E	CGGGGTACCGGGCGGGACCAGGACAA	*Kpn*I	209
	CCGCTCGAGTCCTTCCTCACCACGTGACG	*Xho*I	
*ADAM10*-E1	CGGGGTACCAGCTCTCCGCCGGCG	*Kpn*I	168
	CCGCTCGAGTCCTTCCTCACCACGTGACG	*Xho*I	
*ADAM10*-E2	CGGGGTACCGCGGCGGCAGGCCTA	*Kpn*I	145
	CCGCTCGAGTCCTTCCTCACCACGTGACG	*Xho*I	
*ADAM10*-F	CTGGCCTAACTGGCCGGTACCAGGCGGAGGTCTGAGTTT	*Kpn*I	271
	GCCAGATCTTGATATCCTCGAG	*Xho*I	

### Real-Time Quantitative PCR

Total RNA from SHY5Y or HEK293 cells was extracted using RNAiso plus (Takara, Dalian, Liaoning, China) according to manufacturer’s instructions. cDNA was synthesized from 500 ng total RNA using 5× HiScript II Select qRT Super Mix II (R233-01-AC, Vazyme, Nanjing, China) according to manufacturer’s guidelines. PCR reactions were performed with AceQ qPCR SYBR Green Master Mix (Q111-02, Vazyme, Nanjing, China). The reaction mixture consisted of 0.4 μl of each primer, 4 μl diluted cDNA, 5.2 μl DNase/RNase-free water, and 10 μl 2× SYBR. The parameters were as follows: 95°C for 5 min, 40 cycles at 95°C for 10 s, and 60°C for 30 s. A melting curve was run after each RT-PCR. The Ct value of each sample was calculated, and the relative mRNA level of ADAM10 was normalized relative to that of GAPDH. Fold changes were quantified using the 2^−ΔΔCt^ method. ADAM10 primer sequences (NM_001110) were 5’-TTATGTGCCCCGTGTTCCCTGTTCT-3’ (forward) and 5’-GGTCGAGCCTCCTAGCCTTGATTGG-3’ (reverse). GAPDH primer sequences (NM_001289746) were 5’-CAGGAGGCATTGCTGATGAT-3’ (forward) and 5’-GAAGGCTGGGGCTCATTT-3’ (reverse).

### Cell Viability Assay

Cell viability was measured with a CCK-8 Cell Counting Kit (Vazyme, Nanjing, China) according to manufacturer’s guidelines. HEK293 or SHY5Y cells were seeded on 96-well plates at a density of 1 × 10^3^ or 5 × 10^3^ and cultured overnight; the cells were then treated with different concentrations of cosmosiin or same volume of DMSO for 36 h. Optical density values were determined at 450 nm with Spectra Max 340 PC (Molecular Devices, Sunnyvale, CA, USA).

### Enzyme-Linked Immunosorbent Assay

Culture media were collected after drug treatment was conducted for 36 h. Samples were centrifuged at 14,000 *g* for 10 min at 4°C before analysis. The concentration of human Aβ1–40 or Aβ1–42 was measured using an enzyme-linked immunosorbent assay (ELISA) kit (Cusabio, Wuhan, Hubei, China) according to manufacturer’s guidelines. Absorbance was detected at 450 nm with Spectra Max 340 PC (Molecular Devices, Sunnyvale, CA, USA).

### Statistical Analyses

The experimenters were not blinded to experimental conditions. All data were presented as mean ± SD from at least three independent experiments. Statistical analysis was performed with Prism software (GraphPad Software, La Jolla, CA, USA). Data were analyzed by independent Student’s *t* test or one-way analyses of variance (ANOVA) with a Dunnett’s multiple comparison test. Differences were considered to be significant when *P* < 0.05.

## Results

### Cosmosiin Proportionally Increases the Immature and Mature Forms of ADAM10 Protein Without Affecting the Level of ADAM10 mRNA

**Figure 1:**
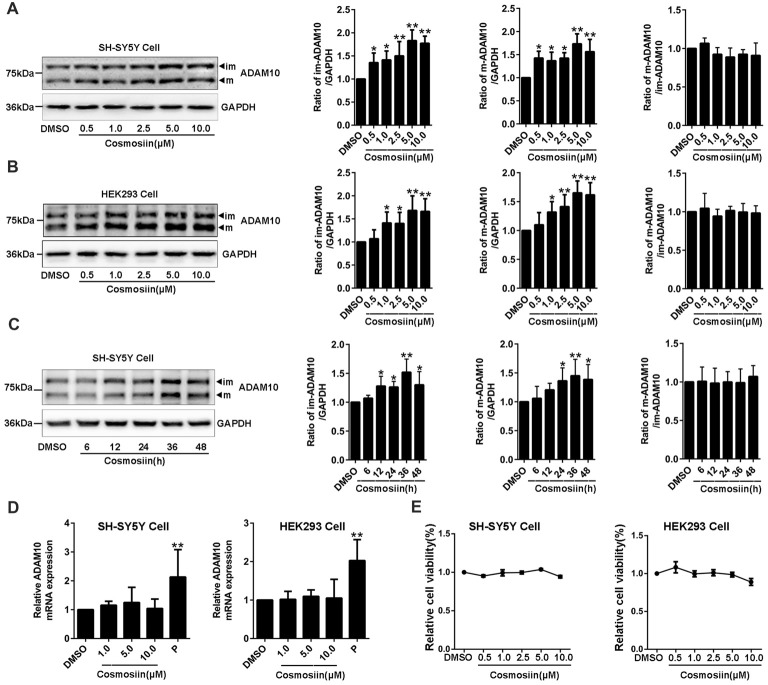
Cosmosiin increases the levels of ADAM10 protein without altering its mRNA level.** (A)** Dose-response effect of cosmosiin on the levels of ADAM10 protein in SH-SY5Y cells. Representative Western blots (left) showing a band near 80 kD and a band near 60 kD, representing the immature (im) and mature (m) forms of ADAM10, respectively; procedures were conducted using SH-SY5Y cells treated with cosmosiin (at 0.5, 1, 2.5, 5 and 10 μM) for 36 h (left). Bar plot summaries of im- and m-ADAM10 protein levels, and m-ADAM10/im-ADAM10 ratio, in the presence of cosmosiin (on the right). **(B)** Dose-response effect of cosmosiin on the levels of ADAM10 protein in HEK293 cells. Representative Western blots (left) and bar plot summaries (right) of the im-ADAM10 and m-ADAM10 protein levels, and m-ADAM10/im-ADAM10 ratio, in HEK293 cells treated with cosmosiin (0.5, 1, 2.5, 5 and 10 μM) for 36 h. **(C)** Time-course effect of cosmosiin on the levels of ADAM10 protein. Representative Western blots (left) and bar plot summaries (right) of the m-/im-ADAM10 in SH-SY5Y cells treated with 5 μM cosmosiin for indicated times (6, 12, 24, 36 and 48 h). **(D)** Relative ADAM10 mRNA levels in SH-SY5Y (left) and HEK293 (right) cells treated with cosmosiin (1, 5 and 10 μM) and apicidin (P, 0.25 μM) for 36 h. **(E)** Relative cell viability of SH-SY5Y (left) and HEK293 (right) cells treated with cosmosiin for 36 h. Data are expressed as means ± SD. *P < 0.05; **P < 0.01 (*n* = 4–5).

To determine whether the small molecule cosmosiin (Supplementary Figure S1) can affect the expression of ADAM10 protein, we first assessed the levels of ADAM10 protein in SH-SY5Y cells treated with cosmosiin. Western blots probing for ADAM10 exhibited two bands near 80 KD and 60 KD (Supplementary Figure S2), which are considered the immature and mature forms of ADAM10, respectively (Woods and Padmanabhan, 2013). Cosmosiin at all concentrations (0.5, 1, 2.5, 5 and 10 μM) significantly increased the levels of immature (im-ADAM10) and mature (m-ADAM10) ADAM10 protein in SH-SY5Y cells (Figure 1A). The ratio of m-ADAM10 to im-ADAM10 was not changed by cosmosiin (Figure 1A), suggesting that cosmosiin did not affect the maturation of ADAM10. Similarly, HEK293 cells also showed a proportional increase in the levels of im-ADAM10 and m-ADAM10 protein after treatment with cosmosiin at 1, 2.5, 5 and 10 μM; the ratio of m-ADAM10 / im-ADAM10 was not significantly altered (Figure 1B). Time-course experiments showed that the enhancement of ADAM10 protein levels started at 12 h and lasted for up to 48 h in SH-SY5Y cells treated with cosmosiin, while the ratio of m-ADAM10 / im-ADAM10 remained unchanged (Figure 1C). To determine whether cosmosiin can influence the transcription of ADAM10, we assessed the level of ADAM10 mRNA in SH-SY5Y and HEK293 cells. We have previously reported that the histone deacetylase inhibitor apicidin can increase the levels of ADAM10 mRNA (Hu X. T. et al., 2017). Thus, apicidin (P, 0.25 μM) was used as positive control. As shown in Figure 1D, while apicidin (P) significantly increased the levels of ADAM10 mRNA, cosmosiin (at 1, 5 and 10 μM) did not significantly alter the levels of ADAM10 mRNA in SH-SY5Y and HEK293 cells. Further cell viability analyses revealed that cosmosiin (at 0.5, 1, 2.5, 5 and 10 μM) was not toxic to cells (Figure 1E). To explore the potential mechanisms of cosmosiin regulation of ADAM10, 5 μM cosmosiin was chosen as the treatment dose for further experiments. Taken together, our results indicate that cosmosiin proportionally increased the levels of ADAM10 protein while the levels of ADAM10 mRNA were not affected in human cell lines. This suggests that the increased levels of ADAM10 protein, induced by cosmosiin, did not occur via ADAM10 maturation or transcriptional regulation.

### Cosmosiin Enhancement of ADAM10 Protein Levels Is Dependent on the 5’UTR of ADAM10

**Figure 2:**
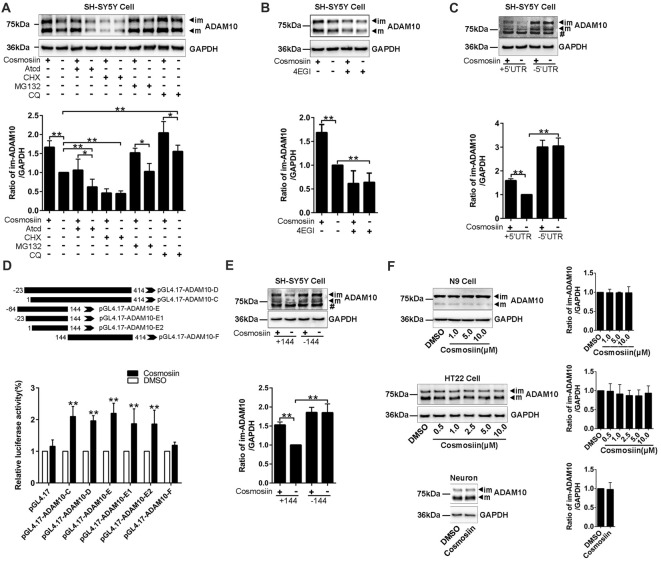
Cosmosiin regulation of ADAM10 involves the first 144 nucleotides of 5’-untranslated region (5’UTR). **(A)** Representative Western blots (top) and bar plot summary (bottom) of ADAM10 levels in SH-SY5Y cells treated with cosmosiin (5 μM, 36 h) or DMSO (1:10,000), in the absence or presence of transcription inhibitor actinomycin D (ActD, 0.1 μM for 12 h), translation inhibitor cycloheximide (CHX, 5 μM for 6 h), proteasome inhibitor MG132 (1 μM for 6 h), or lysosome inhibitor chloroquine (CQ, 100 μM for 6 h). **(B)** Representative Western blots (top) and bar plot summary (bottom) of ADAM10 levels in SH-SY5Y cells treated with cosmosiin (5 μM) in the absence or presence of 4EGI1 (25 μM, 6 h). **(C)** The levels of ADAM10 protein in SH-SY5Y cells transiently transfected with truncated human ADAM10 with deleted 5’UTR (−5’UTR) or with the entire human ADAM10 containing 5’UTR (+5’UTR). ADAM10 levels are significantly reduced in cells containing +5’UTR relative to those in cells with −5’UTR. In −5’UTR transfected cells, there is no significant difference in the levels of ADAM10 between cosmosiin- and control-treated cells. # indicates a nonspecific band. **(D)** Top: The 5’UTR of ADAM10 was truncated into different fragments and subcloned into a pGL4.17 vector to construct the luciferase reporter plasmids: pGL4.17-ADAM10-C, pGL4.17-ADAM10-D, pGL4.17-ADAM10-E, pGL4.17-ADAM10-E1, pGL4.17-ADAM10-E2 and pGL4.17-ADAM10-F. Numbers indicate the relative positions with respect to nucleotides in the 5’UTR. Bottom: relative luciferase activities in SH-SY5Y cells transiently transfected with different pGL4.17ADAM10 plasmids and treated with 5 μM cosmosiin for 36 h. The measured activity of firefly luciferase normalized to the luciferase activity of the internal control plasmid pGL4.17.** (E)** Representative Western blots (top) and bar plot summary (bottom) of 6× His tagged ADAM10 in SH-SY5Y cells transiently transfected with truncated human ADAM10 with the first 144 nucleotides of 5’UTR deleted (−144) or with the entire human ADAM10 containing full-length 5’UTR (+144). # indicates a nonspecific band. **(F)** Representative Western blots (left) and bar plot summary (right) of ADAM10 protein levels in N9 cells (top), HT22 cells (middle) and primary hippocampal neurons (bottom), treated with cosmosiin at different concentrations or treated with the vehicle DMSO (1:10,000) for 36 h. Data are expressed as means ± SD. *P < 0.05; **P < 0.01 (*n* = 3–5).

To further identify how cosmosiin controls the expression of ADAM10 protein, we first assessed the levels of the ADAM10 protein after treatment with transcription inhibitor ActD (0.1 μM for 12 h), translation inhibitor CHX (5 μM for 6 h), proteasome inhibitor MG132 (1 μM for 6 h), and lysosome inhibitor CQ (100 μM for 6 h; Chen et al., 2014; Zhan et al., 2016; Hu L. T. et al., 2017). Under basal conditions, both ActD and CHX significantly decreased the levels of the ADAM10 protein. However, cosmosiin-induced enhancement of the levels of ADAM10 was diminished only in the presence of CHX (translation inhibitor) but not in the presence of ActD (transcription inhibitor; Figure 2A). CQ alone, but not MG132 alone, significantly increased the levels of ADAM10 under basal conditions; this is consistent with the report showing that the lysosomal system was involved in ADAM10 degradation (Maurer et al., 2015). In the presence of MG132 or CQ, cosmosiin-induced enhancement of the levels of ADAM10 was retained (Figure 2A). These results indicate that a translational mechanism was involved in cosmosiin regulation of ADAM10; this was unrelated to transcription and protein degradation mediated by proteasomal or lysosomal systems. Indeed, the translation inhibitor 4EGI1 (at 25 μM for 6 h), which blocks cap-dependent translation (Moerke et al., 2007; Bitterman and Polunovsky, 2012), caused a significant reduction in the levels of ADAM10; 4EGI1 further diminished the effect of cosmosiin on ADAM10 in SH-SY5Y cells (Figure 2B).

To test whether the 5’UTR is involved in cosmosiin regulation of ADAM10, we assessed the effect of cosmosiin on the ADAM10 protein in SH-SY5Y cells. These cells overexpress constructs in which the full-length 5’UTR was included (+5’UTR) or deleted (−5’UTR; Lammich et al., 2010). As shown in Figure 2C, the absence of 5’UTR drastically increased the levels of ADAM10 protein. This effect was not observed with full-length 5’UTR, indicating that the 5’UTR suppresses the translation of ADAM10. In cells overexpressing −5’UTR, the enhancement was blocked by the increase in the levels of ADAM10 occurring from a lack of 5’UTR. These results indicate that the effect of cosmosiin on ADAM10 was critically dependent on the 5’UTR of ADAM10.

To further validate that the 5’UTR is involved in cosmosiin regulation of ADAM10, we performed a translation luciferase assay in SH-SY5Y cells transiently transfected with plasmids containing different lengths of 5’UTR sequences corresponding to the promoter regions: −23 to 414 (−467/−30, pGL4.17-ADAM10-D), 1 to 414 (−44/−30, pGL4.17-ADAM10-C), −64 to 144 (−508/−300, pGL4.17-ADAM10-E), −23 to 144 (−467/−300, pGL4.17-ADAM10-E1), 1 to 144 (−444/−300 pGL4.17-ADAM10-E2), and 144 to 414 (−300/−30, pGL4.17-ADAM10-F). This was performed in the absence and presence of 5 μM cosmosiin for 36 h. As shown in Figure 2D, all fragments of the 5’UTR, except fragment 144–414, showed a significant increase of luciferase activity after cosmosiin treatment, with the minimum length being 1–144. To validate that the first 144 nucleotides of the 5’UTR is involved in cosmosiin regulation of ADAM10, we assessed the effect of cosmosiin on SH-SY5Y cells transfected with the 5’UTR constructs in which the 1–144 fragment was included (+144) or deleted (−144). As shown in Figure 2E, the levels of the ADAM10 protein were increased in −144-expressing cells relative to those in +144-expressing cells (basal conditions), indicating that the first 144 nucleotides of the 5’UTR suppress the translation of ADAM10. Cosmosiin also increased the levels of ADAM10 in +144- but not in −144-expressing cells. These results indicate that cosmosiin regulation of ADAM10 was critically dependent on the first 144 nucleotides of the 5’UTR.

Validating the effect of cosmosiin on murine cells would provide basis for further experiments in mouse models of AD. Therefore, we assessed whether cosmosiin can also increase the levels of the ADAM10 protein in these cells. Surprisingly, cosmosiin failed to increase the levels of the ADAM10 protein in two murine cell lines (N9 and HT22) and in primary cultures of hippocampal neurons (Figure 2F). Thus, we analyzed the 5’UTR sequences of human and mouse ADAM10, revealing that the first 194 nucleotides were lacking in mouse ADAM10 (Supplementary Figure S3). These results also indicate that the first 144 nucleotides of 5’UTR are critical in cosmosiin regulation of ADAM10.

**Figure 3:**
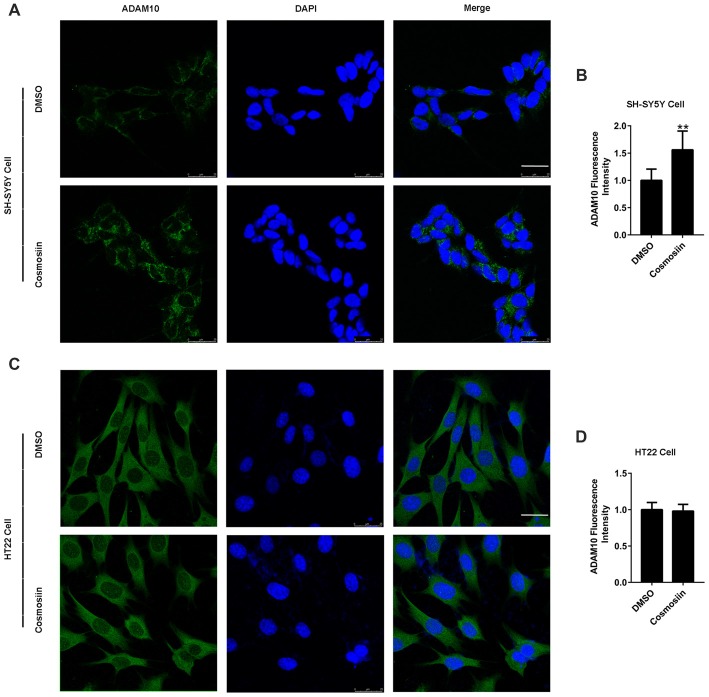
Cosmosiin differentially affects ADAM10 immunofluorescent intensity in SH-SY5Y and HT22 cells.** (A,B)** Representative confocal microscopic images **(A)** and quantification **(B)** of immunofluorescence intensity of ADAM10 (green) in human SH-SY5Y cells in the absence (treated with DMSO only) and presence of cosmosiin (5 μM for 36 h). **(C,D)** Representative confocal microscopic images **(C)** and quantification **(D)** of immunofluorescence intensity of ADAM10 (green) in murine HT22 cells in the absence (treated with DMSO only) and presence of cosmosiin (5 μM for 36 h). DAPI (blue), a nuclear marker, served as internal control. Cosmosiin significantly increases immunofluorescence signals of ADAM10 in SH-SY5Y but not in HT22 cells. Scale bar, 25 μm. **P < 0.01 (*n* = 50).

We used Western blotting to show that cosmosiin differentially regulated the levels of ADAM10 protein in human and murine cells. To further validate this result, we performed immunofluorescence studies. As shown in Figures 3A,B, treatment with cosmosiin significantly increased the immunofluorescence intensity of ADAM10 in SH-SY5Y cells. In contrast, no significant differences in immunofluorescence intensity were found in HT22 cells treated with cosmosiin and control (Figures 3C,D).

### PI3K Signaling Is Involved in Cosmosiin Regulation of ADAM10

**Figure 4:**
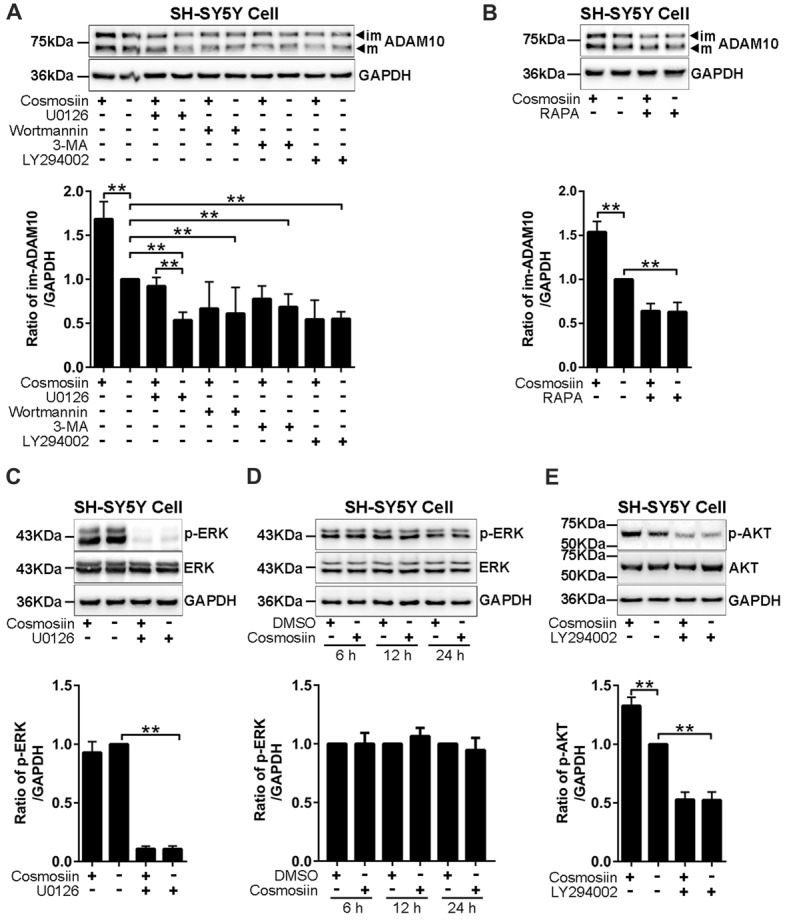
Phosphatidylinositide 3-kinase (PI3K) signaling is involved in cosmosiin regulation of ADAM10.** (A)** Representative Western blots (top) and bar plot summary (bottom) of ADAM10 levels in SH-SY5Y cells treated with cosmosiin (5 μM) for 36 h and pre-incubated for 1 h with: ERK inhibitor U0126 (20 μM); PI3K inhibitors LY294002 (10 μM), wortmannin (0.1 μM), or 3-MA (50 μM). **(B)** Representative Western blots (top) and bar plot summary (bottom) of ADAM10 levels in SH-SY5Y cells treated with cosmosiin (5 μM) for 36 h and pre-incubated for 1 h with mTOR inhibitor rapamycin (RAPA, 5 μM). **(C)** Representative Western blots (top) and bar plot summary (bottom) of ERK and phosphorylated ERK (p-ERK) levels in SH-SY5Y cells in the absence or presence of cosmosiin (5 μM) or U0126 (20 μM) for 36 h. **(D)** Representative Western blots (top) and bar plot summary (bottom) of ERK and phosphorylated ERK (p-ERK) in SH-SY5Y cells treated with the vehicle DMSO (1:10,000) or 5 μM cosmosiin for indicated times (6, 12 and 24 h). **(E)** Representative Western blots (top) and bar plot summary (bottom) of AKT and phosphorylated AKT (p-AKT) in SH-SY5Y cells in the absence or presence of cosmosiin (5 μM) or LY294002 (10 μM) for 36 h. Data are expressed as means ± SD. *P < 0.05; **P < 0.01 (*n* = 3–4).

Translation initiation is a rate-limiting process that is regulated by PI3K signaling (Sonenberg and Hinnebusch, 2009). To test whether this signaling pathway is involved in the cosmosiin regulation of ADAM10, we assessed the levels of the ADAM10 protein in SH-SY5Y cells treated with the following agents: PI3K inhibitors LY294002 (10 μM), wortmannin (0.1 μM), and 3-MA (50 μM); ERK inhibitor U0126 (20 μM); and mTOR inhibitor rapamycin (5 μM; Hu L. T. et al., 2017; Hu X. T. et al., 2017). As shown in Figure 4A, U0126 and the PI3K inhibitors LY294002, wortmannin and 3-MA significantly reduced the basal level of the ADAM10 protein compared with the levels of the control. In cells treated with U0126, cosmosiin increased the levels of the ADAM10 protein from below baseline to near baseline. In the presence of all three PI3K inhibitors, cosmosiin failed to increase the levels of the ADAM10 protein (Figure 4A). Similarly, the mTOR inhibitor rapamycin reduced the basal level of ADAM10 protein and prevented the cosmosiin-induced enhancement of ADAM10 levels (Figure 4B). To further determine whether ERK or PI3K activity is influenced by cosmosiin, we assessed ERK and PI3K phosphorylation in SH-SY5Y cells treated with cosmosiin. As shown in Figure 4C, while U0126 drastically reduced the phosphorylation of ERK (p-ERK), indicating that U0126 is an effective ERK inhibitor, treatment with cosmosiin did not alter the level of p-ERK at 36 h (Figure 4C), or at earlier time points ranging from 6 h to 24 h (Figure 4D). In contrast, cosmosiin caused a significant increase in AKT phosphorylation (p-AKT), which was drastically reduced in the presence of the PI3K inhibitor LY294002 (Figure 4E). These results suggest that MAPK/ERK signaling can affect the levels of ADAM10 protein, but do not explain the cosmosiin-induced increase in the levels of ADAM10. Alternatively, PI3K signaling may play an important role in this process.

### Cosmosiin Increases APP Processing and Reduces Aβ Level

**Figure 5:**
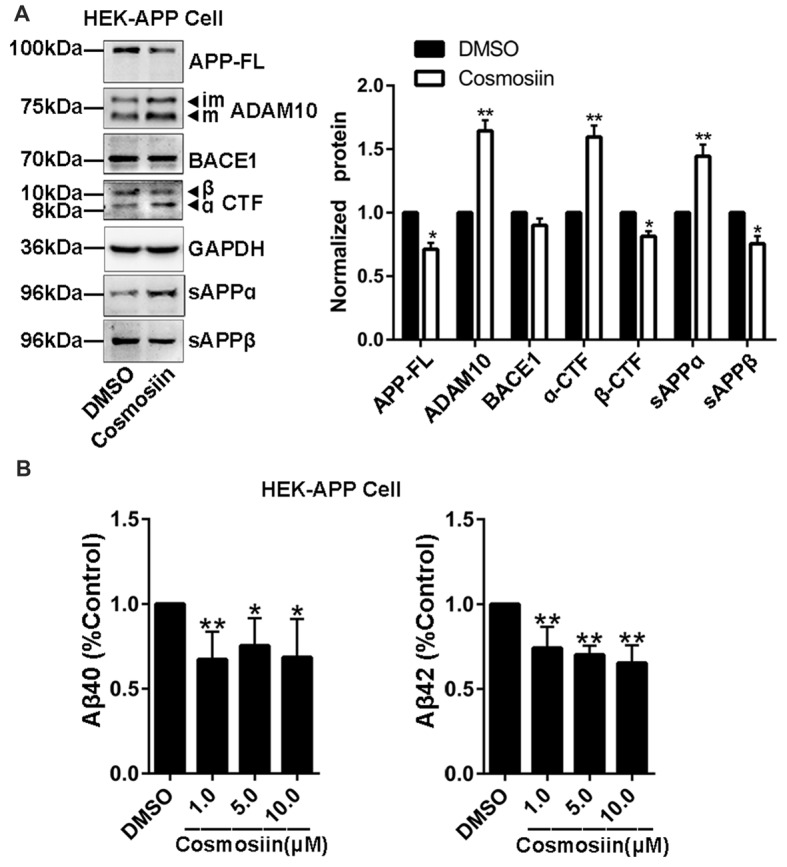
Cosmosiin affects amyloid precursor protein (APP) processing and Aβ production.** (A)** Representative Western blots (left) and bar plot summary (right) of full-length APP (APP-FL), ADAM10, BACE1, α-/β-CTF, sAPPα and sAPPβ levels in HEK-APP cells treated with the vehicle DMSO (1:10,000) or 5 μM cosmosiin for 36 h. **(B)** Normalized levels of Aβ1–40 (left) and Aβ1–42 (right) measured by enzyme-linked immunosorbent assay (ELISA) in the culture medium of HEK-APP cells treated with the vehicle DMSO (1:10,000) or cosmosiin (1, 5 and 10 μM) for 36 h. Data are expressed as means ± SD. *P < 0.05; **P < 0.01 (*n* = 4).

Provided that the upregulation of ADAM10 precludes the generation of neurotoxic Aβ (Singer et al., 2005), the cosmosiin-induced increase in the levels of ADAM10 may also influence APP processing and Aβ levels. Thus, we assessed the effect of cosmosiin on the protein levels of APP, sAPPα and α-CTF in HEK-APP cells that stably express the human full-length APP protein (APP-FL). As shown in Figure 5A, in cells treated with cosmosiin, the levels of APP-FL protein were significantly reduced, while those of sAPPα and α-CTF were significantly increased. Conversely, although cosmosiin did not cause a significant change in the levels of BACE1 protein, the levels of sAPPβ and β-CTF were significantly reduced. Consistently, the results of the ELISA assay showed that the levels of Aβ1–42 and Aβ1–40 were significantly reduced by treatment with cosmosiin at 1, 5 and 10 μM (Figure 5B).

## Discussion

Cosmosiin exhibits anti-oxidant, anti-inflammatory and anti-cancer activities (Fuchs and Milbradt, 1993; Powell et al., 2012; Miguel et al., 2015; Lin and Frost, 2017). The insulin-mimetic action of cosmosiin is associated with diabetic complications (Rao et al., 2011). In the nervous system, cosmosiin shows a protective effect against aging- and drug-induced cognitive impairment and possesses anxiolytic potential (Patil et al., 2003; Kumar and Bhat, 2014). In the present study, we provide evidence that cosmosiin increases the levels of ADAM10 protein in human cell lines.

The flavonoid (-)-epigallocatechin-3-gallate (EGCG) can promote furin-mediated maturation of ADAM10 (Fernandez et al., 2010). Autophagic inhibition leads to increased levels of the ADAM10 protein (Maurer et al., 2015; Powers and Bubeck Wardenburg, 2015). In our study, the proportional enhancement of both m- and im-ADAM10 proteins excludes the possibility that cosmosiin may affect ADAM10 maturation. Although the autophagic inhibitor CQ, but not the proteasomal inhibitor MG132, increases the levels of ADAM10 protein under basal conditions (Maurer et al., 2015), these drugs failed to influence cosmosiin-mediated enhancement of ADAM10. Alternatively, the translation inhibitors CHX and 4EGI attenuated the effect of cosmosiin on ADAM10, supporting the notion that a translational mechanism is involved.

The presence of 5’UTR suppresses the translation of ADAM10 without altering its mRNA level (Lammich et al., 2010). A potential G-quadruplex motif between nucleotides 66 and 94 (−350 to −378) plays an important role in translational suppression of the 5’UTR (Lammich et al., 2011). Interestingly, this G-quadruplex motif can bind 1-methylquinolinium derivatives, which attenuates the 5’UTR-induced suppression of ADAM10 translation and results in increased expression of ADAM10 (Dai et al., 2015). In our study, the involvement of 5’UTR in cosmosiin-mediated regulation of ADAM10 is supported by the following findings. (1) ADAM10 mRNA was not altered in cosmosiin-treated human cells. (2) Cosmosiin-induced enhancement of ADAM10 was diminished in cells overexpressing the 5’UTR-negative construct. (3) Luciferase assay revealed that the first 144 nucleotides (−300 to −444) are necessary for the effect of cosmosiin on ADAM10 expression. In contrast, nucleotides from 144 to 414 (−300 to −30) did not respond to cosmosiin. (4) In murine cells, which lack the first 194 nucleotides (−250 to −444) of the 5’UTR, cosmosiin failed to increase the levels of ADAM10. These results indicate that at least the first 144 nucleotides (−300 to −444) of 5’UTR are critical for cosmosiin-mediated enhancement of ADAM10 expression.

PI3K and ERK signaling have strong effects on translational regulation in eukaryotes (Bjornsti and Houghton, 2004; Holz et al., 2005; Sonenberg and Hinnebusch, 2009). In the nervous system, translation of the synaptic proteins is tightly regulated by PI3K- and ERK-dependent mechanisms (Sutton and Schuman, 2006; Costa-Mattioli et al., 2009). Conversely, cosmosiin is considered an agonist of estrogen receptors (Shanle et al., 2011; Powell et al., 2012), which can activate downstream PI3K and ERK signaling (Mannella and Brinton, 2006; Zhao and Brinton, 2007). Cosmosiin also possesses insulin-mimetic effects, which can enhance the phosphorylation of insulin receptor-β, suggesting that PI3K and ERK are involved in cosmosiin function (van der Heide et al., 2006). In our study, ERK inhibition by U0126 reduced the levels of ADAM10 protein. Similarly, inhibition of PI3K and mTOR (Zhang et al., 2010) also led to decreased basal levels of ADAM10, indicating that both ERK and PI3K are involved in ADAM10 regulation under basal conditions. Our results further show that in the presence of U0126, cosmosiin maintained the levels of ADAM10 at baseline; however, in the presence of PI3K inhibitors, cosmosiin failed to increase the levels of ADAM10. In line with this finding, cosmosiin did not alter ERK activity as measured by p-ERK, but significantly increased PI3K activity as measured by p-AKT. Thus, it appears that PI3K, rather than ERK, is likely involved in the regulation of ADAM10 by cosmosiin. The underlying mechanisms are currently unknown. We have previously reported that the inhibitor of ERK, but not that of PI3K, diminishes apicidin-mediated regulation of ADAM10 transcription (Hu X. T. et al., 2017). It is possible that while ERK activity may be closely associated with transcriptional elements, PI3K and mTOR may play more important roles in ADAM10 translation (Sonenberg and Hinnebusch, 2009); this remains to be clarified in the future. It is also worth noting that these results were obtained using SH-SY5Y cells. The ERK/PI3K signaling identified here may be different in mature neurons.

Importantly, we found that cosmosiin promoted the non-amyloidogenic cleavage of APP, resulting in reduced levels of Aβ. Although cosmosiin did not change the levels of BACE1 protein, the β-site cleavage of APP, as measured by sAPPβ, was decreased by cosmosiin. Overexpression of sAPPα in transgenic mice leads to reduced BACE1 activity (Obregon et al., 2012). Recently, sAPPα was identified as an endogenous inhibitor of BACE1 (Peters-Libeu et al., 2015). Thus, it is possible that the decreased levels of sAPPβ may result from BACE1 inhibition, which is caused by sAPPα.

The limitation of our study is that the ineffectiveness of cosmosiin in murine cells prevented us from assessing whether cosmosiin alleviates cognitive decline in mouse models of AD. In addition, most of the results were obtained using SH-SY5Y cells; these results do not necessarily indicate that cosmosiin increases the levels of ADAM10 protein in postmitotic neurons, especially in the brains of patients with AD. Further studies are needed to investigate this notion.

## Conclusion

Our study revealed that cosmosiin increases the expression of ADAM10 via 5’UTR- and PI3K-dependent mechanisms, suggesting that the natural flavonoid cosmosiin may have therapeutic potential in the treatment of AD.

## Author Contributions

G-JC and ZY designed the study. ZM and YT performed the experiments and analyzed the data. X-TH, B-LZ, Y-LM, J-SZ, and X-JD provided assistance with the research. ZM and G-JC wrote the manuscript.

## Conflict of Interest Statement

The authors declare that the research was conducted in the absence of any commercial or financial relationships that could be construed as a potential conflict of interest.

## Supplementary Material

The Supplementary Material for this article can be found online at: https://www.frontiersin.org/articles/10.3389/fnmol.2018. 00198/full#supplementary-material

FIGURE S1 Chemical structure of cosmosiin.

FIGURE S2 A full blot of ADAM10. Representative Western blots of ADAM10 in a full membrane. Proteins were extracted from SH-SY5Y cells treated with vehicle DMSO (1:10000) or 5 μM cosmosiin for 36 h, and were probed by ADAM10 antibody (ab1997; 1:1000).

FIGURE S3 Sequence alignment between human and mouse ADAM10 5’UTRs. A MegAlign program (DNAstar, Madison, WI, USA) was used to perform alignment analysis between human ADAM10 5’UTR (NCBI Reference Sequence: NM_001110.3, GI:114657268) and mouse ADAM10 5’UTR (NCBI Reference Sequence: NM_007399.4, GI:1025608641). The first 194 nucleotides of ADAM10 5’UTR are lacking in mouse. ATG denotes start codon.

## Abbreviations

α-CTF: α-COOH-terminal fragment
3-MA: 3-methyladenine
5’UTR: 5’-untranslated region
Aβ: accumulation of β-amyloid protein
ActD: actinomycin D
AD: Alzheimer’s disease
ADAM10: a disintegrin and metalloproteinase domain-containing protein
APP: β-amyloid precursor protein
sAPPα: soluble β-amyloid precursor protein α
BACE1: β-amyloid converting enzyme 1
CHX: cycloheximide
CQ: chloroquine
EGCG: (-)-epigallocatechin-3-gallate
ERK: extracellular regulated protein kinases
PI3K: phosphatidylinositide 3-kinase
RIPA: radio immunoprecipitation Assay
sAPPα: soluble β-amyloid precursor protein α
SP1: specific protein 1
USF1: upstream transcription factor 1
