# Supplementary figures and images for: Cosmosiin Increases ADAM10 Expression via Mechanisms Involving 5’UTR and PI3K Signaling
Source: Front Mol Neurosci. 2018 Jun 11;11:198. doi: 10.3389/fnmol.2018.00198 (PMC6004422; doi:10.3389/fnmol.2018.00198)

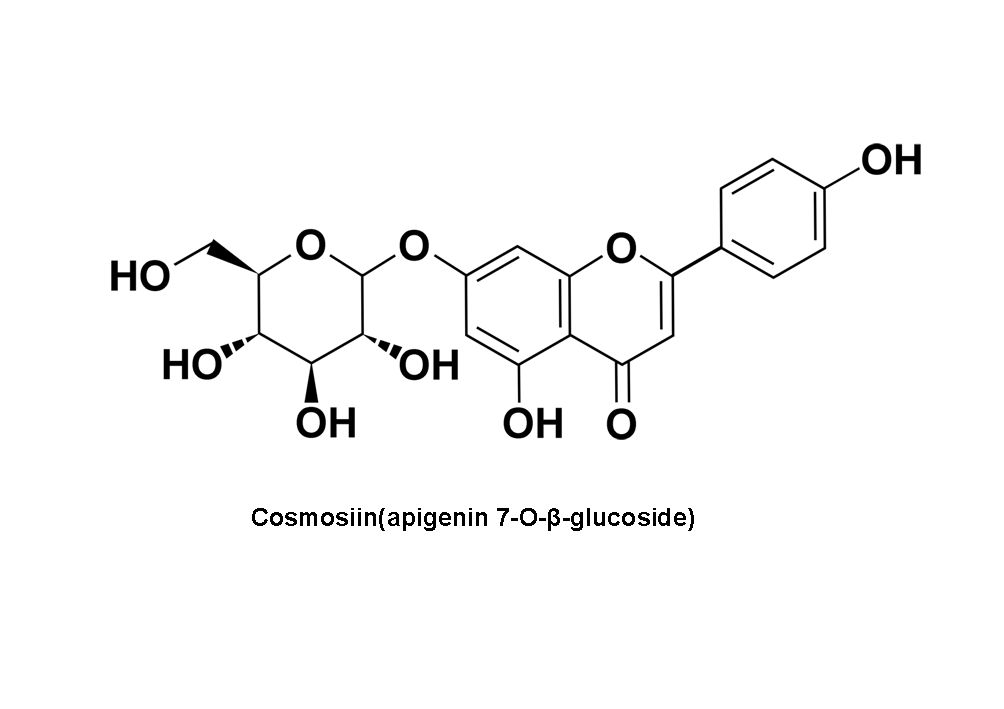

Supplement: FIGURE S1 — Chemical structure of cosmosiin. [file Image_1.TIF]

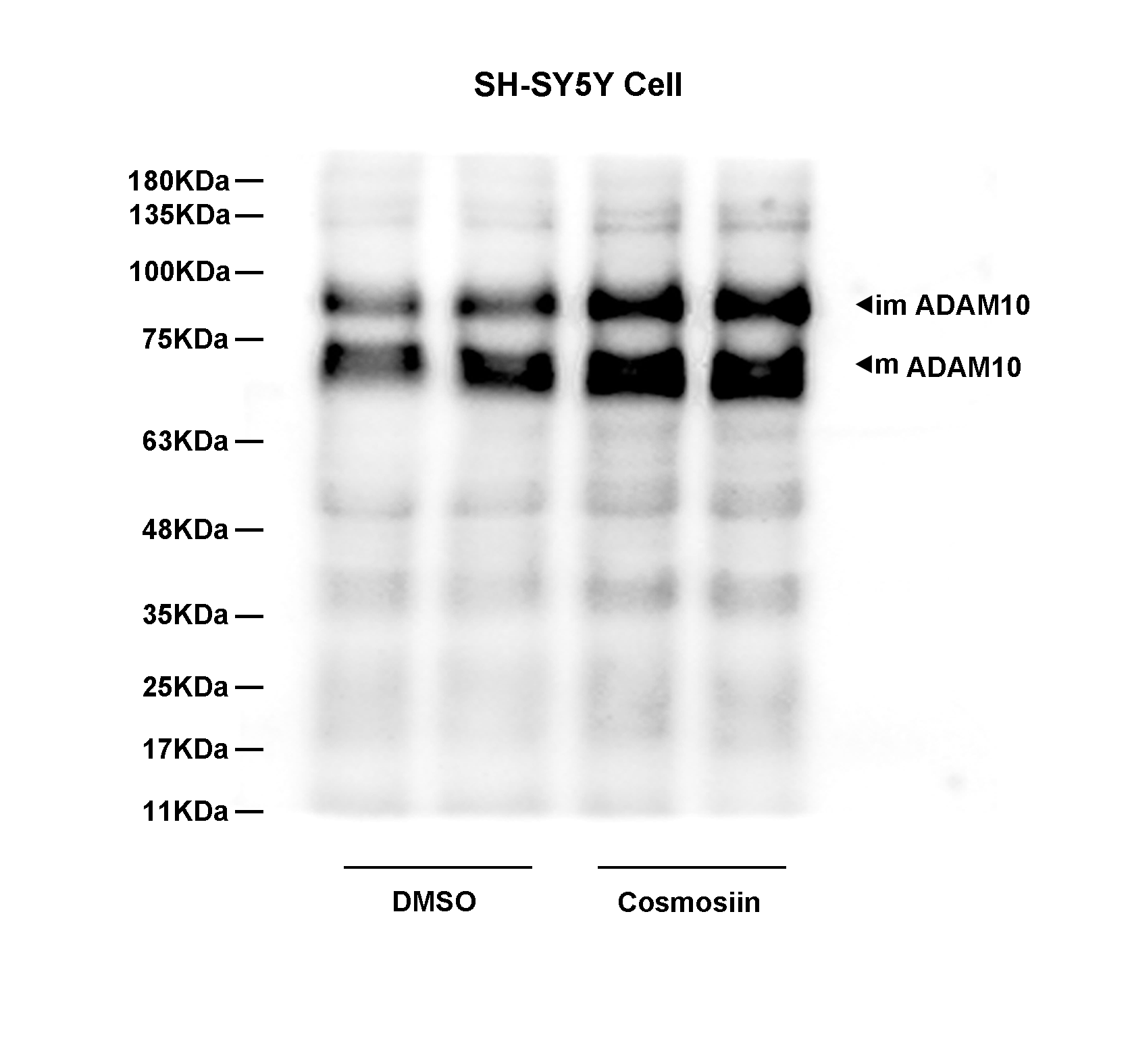

Supplement: FIGURE S2 — A full blot of ADAM10. Representative Western blots of ADAM10 in a full membrane. Proteins were extracted from SH-SY5Y cells treated with vehicle DMSO (1:10000) or 5 μM cosmosiin for 36 h, and were probed by ADAM10 antibody (ab1997; 1:1000). [file Image_2.TIF]

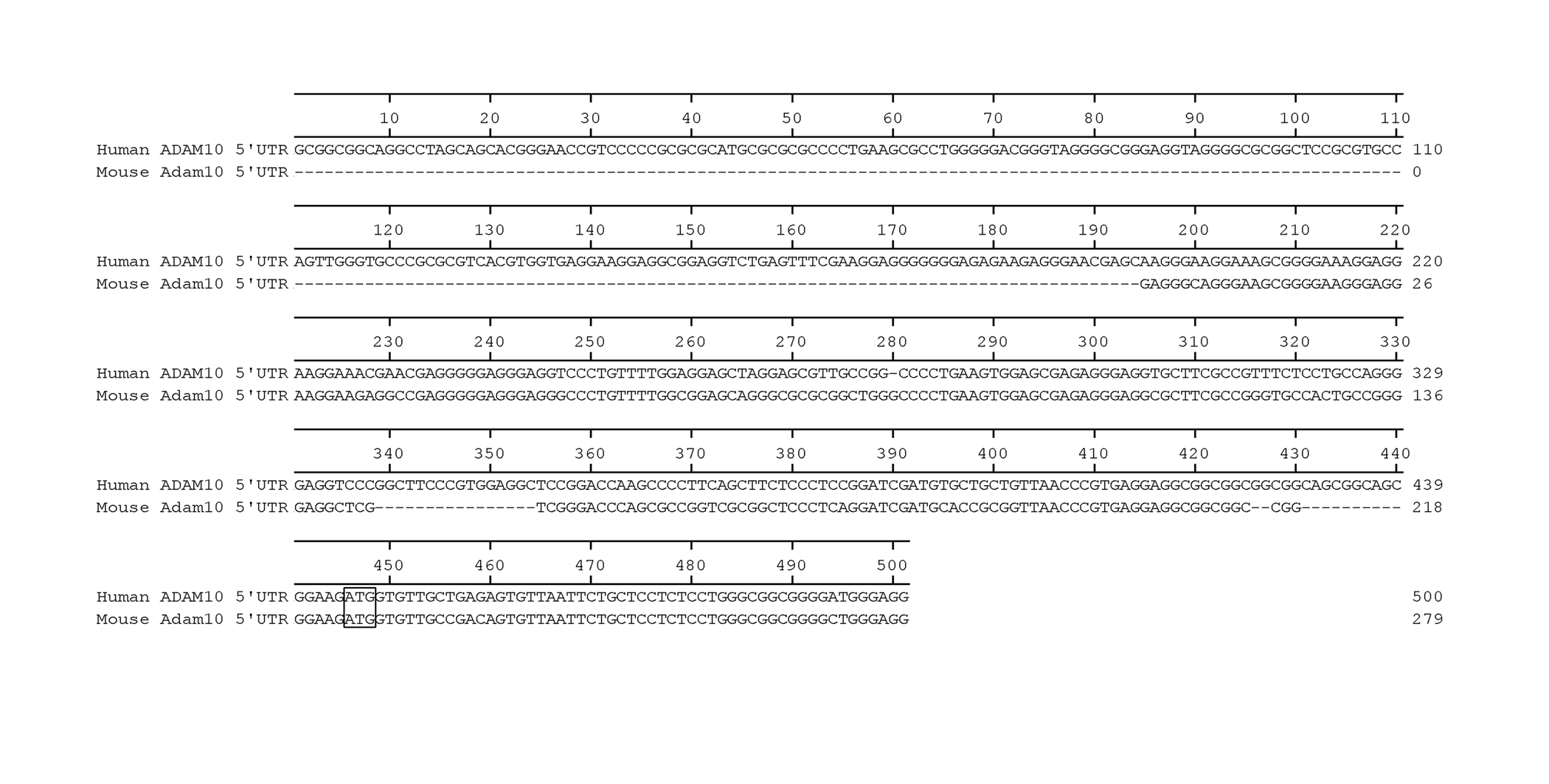

Supplement: FIGURE S3 — Sequence alignment between human and mouse ADAM10 5’UTRs. A MegAlign program (DNAstar, Madison, WI, USA) was used to perform alignment analysis between human ADAM10 5’UTR (NCBI Reference Sequence: NM_001110.3, GI:114657268) and mouse ADAM10 5’UTR (NCBI Reference Sequence: NM_007399.4, GI:1025608641). The first 194 nucleotides of ADAM10 5’UTR are lacking in mouse. ATG denotes start codon. [file Image_3.TIF]
